# Lactation Consultant Access and Breastfeeding Outcomes in the United States: Cross-Sectional Analysis

**DOI:** 10.2196/70098

**Published:** 2025-07-17

**Authors:** James F Dockins, Heather D Pahl, David J Lingerfelt

**Affiliations:** 1 Rockhurst University, 1100 Rockhurst Road, 300 Conway Hall, Kansas City, MO, 64110, United States, 1 816 889 8906; 2 University of Arkansas – Fort Smith, Fort Smith, AR, United States

**Keywords:** breastfeeding, lactation consultant, exclusive breastfeeding, artificial baby milk

## Abstract

**Background:**

Breastfeeding provides unmatched health, developmental, and economic benefits to both infants and mothers, yet breastfeeding continuation rates remain suboptimal in the United States, especially beyond the early postpartum period. Despite well-documented advantages, many mothers face challenges that lead to early cessation, including lack of access to skilled lactation support. International Board Certified Lactation Consultants (IBCLCs) are considered the clinical gold standard in lactation care, but their availability varies widely across states. Understanding how IBCLC access relates to breastfeeding outcomes at the population level is critical to informing equitable public health interventions.

**Objective:**

The aim of this study is to determine whether state-level IBCLC density is associated with breastfeeding initiation and exclusive breastfeeding at 3 and 6 months.

**Methods:**

This cross-sectional analysis used publicly available 2022 data from the Centers for Disease Control and Prevention, US Census Bureau, and the International Board of Lactation Consultant Examiners. IBCLC density per 100,000 women of childbearing age (15‐49 years) was calculated for each of the 50 US states. Breastfeeding outcome data included initiation, exclusive breastfeeding at 3 months, and exclusive breastfeeding at 6 months. Simple and multiple linear regressions were conducted to evaluate the association between IBCLC density and breastfeeding outcomes, adjusting for income, education, and insurance coverage.

**Results:**

IBCLC density ranged from 14.4 to 60.7 per 100,000 women of childbearing age across US states, with a national average of 25.5. Pearson correlation analysis indicated significant positive associations between IBCLC density and breastfeeding outcomes, including initiation (*r*=0.38; *P*<.001), exclusive breastfeeding at 3 months (*r*=0.52; *P*<.001), and exclusive breastfeeding at 6 months (*r*=0.32; *P*<.001). In multiple linear regression models adjusting for income, education, and insurance status, IBCLC density remained significantly associated with all 3 outcomes. For breastfeeding initiation, the adjusted β was 0.26 (95% CI 0.08-0.44; *P*=.005); for exclusive breastfeeding at 3 months, β was 0.43 (95% CI 0.23-0.63; *P*<.001); and for exclusive breastfeeding at 6 months, β was 0.25 (95% CI 0.12-0.39; *P*<.001). Adjusted *R*² values for the models ranged from 0.42 to 0.44, indicating moderate explanatory power.

**Conclusions:**

Higher IBCLC density is significantly associated with improved breastfeeding outcomes at the state level, particularly exclusive breastfeeding at 3 months. These findings support initiatives to expand access to professional lactation support as part of public health strategies to improve breastfeeding rates.

## Introduction

### Background

Breastfeeding is a complex, symbiotic process between a mother and child and breast milk is the one source of infant nutrition that is perfectly, biologically suited for an infant in its unadulterated form. The value of breastfeeding goes well beyond meeting nutritional needs and it is a mother’s way to continue her uniquely profound role of nourishing and nurturing after childbirth. Although breastfeeding is the biological norm for humans, it is no longer the social norm. With the early 20th century commercialization of infant formula, which produces over US $55 billion in sales annually, mothers have been dissuaded from breastfeeding [1]. Infant formula marketing is partially to blame for the decrease in breastfeeding on a global scale—a drop from 90% in the 20th century to 42% in the 21st century [2]. Infant formula usage increasingly became the social norm while mothers increasingly lost their innate ability to breastfeed. Mothers have fewer supporters of breastfeeding as fewer people breastfeed, including their role models and peers. Although breastfeeding is innate, it can be incredibly challenging, especially considering most mothers today do not grow up seeing others breastfeed regularly. The potential problems that mothers can encounter are numerous and often lead to a mother discontinuing breastfeeding prematurely [3].

In the United States, approximately 83% of infants have ever breastfed [4]. This figure even includes babies who have latched just once in their life after birth, but were never breastfed again. By 3 months, the proportion of infants exclusively breastfed drops to approximately 45%, and then to 25% at 6 months. Nearly one-fifth of breastfed infants have already been given infant formula (artificial baby milk) before they are 2 days old. In a study by Perrine et al [5], approximately 68% of the participants did not meet their intended breastfeeding goal. The overarching message is that most women are breastfeeding for shorter durations than they intended.

Lactation consultants are clinical health care professionals that make it their mission to help mothers meet their breastfeeding goals and make breastfeeding the social norm again.

Lactation consultants are extensively educated and trained to understand the biological, anatomical, physiological, psychological, and social mechanisms of breastfeeding—on the part of both the mother and the infant. They are typically employed by hospitals or pediatric clinics or work in private practice. Lactation consultants are effective at improving breastfeeding outcomes, including exclusivity, duration, self-efficacy, and maternal mental health [6,7]. Although lactation consultants do indeed solve many breastfeeding problems, the problem of access to care remains. The lactation consultant profession is fairly small (approximately 19,000 in the United States and 35,000 worldwide) but growing, and it can be difficult for a mother to have geographical access to a lactation consultant [8].

### Research Questions and Hypotheses

This study aims to evaluate the relationship between the population density of lactation consultants and breastfeeding outcomes across US states.

Research question: Is there a correlation between the population density of lactation consultants and breastfeeding outcomes?

The H₀ (null hypothesis) is that there is no correlation between the population density of lactation consultants and breastfeeding outcomes. The H₁ (alternative hypothesis) is that there is a correlation between the population density of lactation consultants and breastfeeding outcomes.

### Study Rationale

Given that breastfeeding rates nationally and globally are low, especially for continuation of breastfeeding, the focus of this article examines access to lactation consultants, who are designated clinical breastfeeding professionals. Access to lactation consultants is a topic that has not been well researched. In the United States, postnatal support varies significantly depending on geographic location, health care provider networks, and insurance status. Unlike some countries with nationalized health care systems, the United States lacks a standardized postnatal care pathway, and support for breastfeeding often depends on fragmented services offered through hospitals, outpatient clinics, and community health programs. However, there is research indicating that the use of lactation consultants does contribute to better breastfeeding outcomes, including longer duration and fewer problems. The inclusion of key sociodemographic variables in this study—household income, education level, and health insurance coverage—reflects their importance as social determinants of health. These factors can significantly influence a mother’s ability to access lactation support services. For example, lower-income mothers or those without insurance may face barriers such as out-of-pocket costs or limited availability of lactation consultants in their communities. Similarly, differences in educational attainment may affect awareness of available resources and breastfeeding best practices. One gap in the current literature is an examination of the relationship between the access to lactation consultants and breastfeeding outcomes. Access to lactation consultants is a different problem entirely than the effectiveness of lactation consultants.

The objective of this study is to examine access to lactation consultants in the United States by measuring their population density at the state level and analyzing its association with key breastfeeding outcomes.

Subsequently, if the results indicate a higher population density of lactation consultants (International Board Certified Lactation Consultants [IBCLCs]) in an area results in better breastfeeding rates, a case can be made for public policy and funding to support improved access to lactation consultants.

## Methods

### Study Design and Setting

This study used a cross-sectional ecological design to investigate associations between state-level availability of lactation consultants and breastfeeding outcomes across the 50 US states. Data were compiled from publicly available national sources representing the year 2022. No human participants were recruited, as the analysis was based on aggregated secondary data. The setting spanned all 50 US states, and data collection periods correspond to annual releases from the US Census Bureau, the Centers for Disease Control and Prevention (CDC), and the International Board of Lactation Consultant Examiners (IBLCE).

### Participants and Eligibility

The unit of analysis was each individual US state. All 50 states were eligible and included in the study based on the availability of complete data across key variables. The District of Columbia and US territories were excluded to ensure consistency in health care infrastructure, population metrics, and reporting standards. As an ecological study, eligibility criteria were based on data completeness at the state level rather than individual characteristics.

### Data Sources and Assessments

Workforce statistics for IBCLCs were obtained from IBLCE’s published reports, which detail the number of actively certified professionals per state. IBCLC density, the primary exposure variable, was calculated as the number of IBCLCs per 100,000 women aged 15-49 years using 2022 US Census Bureau data [9,10]. Breastfeeding outcomes were obtained from the CDC’s 2022 Breastfeeding Report Card, which draws on data from the National Immunization Survey. The 3 key outcomes assessed were breastfeeding initiation (defined as ever having breastfed), exclusive breastfeeding at 3 months, and exclusive breastfeeding at 6 months. These outcomes represent population-level estimates based on parent-reported data and are considered valid for surveillance purposes.

The Breastfeeding Report Card data are derived from the National Immunization Survey, a telephone-based survey of parents with children aged 19-35 months. The CDC uses standardized weighting methods to ensure national representativeness. Although this is a well-established source of breastfeeding surveillance data, it is important to acknowledge that self-reported data can be subject to recall and social desirability bias.

Additionally, the study used the World Health Organization’s definition of women of reproductive age—15 to 49 years old—to calculate IBCLC density [11]. This aligns with global demographic standards and ensures comparability across public health data sources.

The study also included 3 covariates identified as potential confounders based on existing literature about social determinants of health: average household income, the percentage of the population without health insurance, and the percentage of women aged 25 years and older with a bachelor’s degree or higher. These variables were extracted from the US Census Bureau’s 2022 state-level data. Together, these measures were used to assess sociodemographic context and its potential influence on breastfeeding behaviors.

### Study Size

The final study population included all 50 US states. This sample size was determined by the availability of complete and consistent state-level data for all variables of interest. As a fixed and exhaustive sample of all states, a formal power analysis or sample size calculation was not necessary or applicable to this ecological analysis.

### Data Analysis

Descriptive statistics were used to summarize the distribution of IBCLC density and breastfeeding outcomes across states. Simple linear regression models were conducted to assess the unadjusted associations between IBCLC density and each of the 3 breastfeeding outcomes. Subsequently, multiple linear regression models were developed to control for the influence of income, insurance coverage, and educational attainment. The primary independent variable in all models was IBCLC density, while the dependent variables were breastfeeding initiation, exclusive breastfeeding at 3 months, and exclusive breastfeeding at 6 months.

Pearson correlation analyses were conducted to examine the strength and direction of linear relationships between IBCLC density and each of the 3 breastfeeding outcomes: initiation, exclusive breastfeeding at 3 months, and exclusive breastfeeding at 6 months. These analyses provided initial insight into bivariate associations and guided the selection of variables for subsequent regression modeling. Correlation coefficients (*r*) and *P* values were reported to assess statistical significance.

Regression results were reported as standardized beta coefficients (β) along with 95% CIs, *P* values, and adjusted *R*² values to assess statistical significance and explanatory power. All analyses were conducted using Python programming tools (version 3.11; Python Software Foundation).

### Ethical Considerations

This research involved the secondary analysis of publicly available, deidentified datasets and did not include any interaction with human subjects or use of private, identifiable information. Accordingly, ethics board review was not sought, in accordance with US Department of Health and Human Services regulations (45 CFR 46.102), which state that such studies do not constitute human subjects research. Institutional policy at Rockhurst University confirms that publicly available and anonymized data analyses are exempt from institutional review board review. All data used in this study were obtained from official public sources, including the CDC, US Census Bureau, and IBLCE. The study adhered to the STROBE (Strengthening the Reporting of Observational Studies in Epidemiology) guidelines for transparent reporting of observational cross-sectional research.

### Statistical Testing

To enable consistent cross-state comparisons, the researchers first calculated the density of IBCLCs per 100,000 females of childbearing age (15‐49 years) using Microsoft Excel 2021 (Microsoft Corp).

Simple linear regression analyses were conducted in Python to evaluate the unadjusted relationship between IBCLC density and each of the 3 key breastfeeding outcomes:

1. Breastfeeding initiation.
2. Exclusive breastfeeding at 3 months.
3. Exclusive breastfeeding at 6 months.

Multiple linear regression was then used to control for potential confounding variables. The dependent (outcome) variables remained the same: breastfeeding initiation, exclusive breastfeeding at 3 months, and exclusive breastfeeding at 6 months. The independent variables in the adjusted models included:

- IBCLC population density (primary exposure)
- Average household income
- Proportion of the population without health insurance
- Proportion of the population with a bachelor’s degree or higher

These covariates were selected based on their theoretical importance as social determinants of health, supported by prior research linking these factors to maternal and infant health disparities [12]. Variables were added to the regression models simultaneously; no stepwise (forward or backward) selection methods were applied.

For each model, we report regression coefficients, *P* values, and adjusted *R²* values to assess the strength, significance, and explanatory power of the observed relationships.

For reader clarity, we have included definitions of relevant terms in Multimedia Appendix 1.

## Results

The relevant demographic makeup for each state is shown in Table 1, along with measures for the United States on average.

The breastfeeding initiation, exclusivity at 3 months, and exclusivity at 6 months rates for each state and the United States averages are shown in Table 2.

**Table 1.** Demographics by state.

State	Female childbearing population, n	Income, US $	Bachelor’s or higher, %	No health insurance, %
Alabama	1,143,118	59,674	28.8	8.8
Alaska	163,121	88,121	30.6	11
Arizona	1,637,098	74,568	33	10.3
Arkansas	677,271	55,432	25.4	8.4
California	9,124,204	91,551	37	6.5
Colorado	1,380,866	89,302	45.9	7.1
Connecticut	804,136	88,429	41.9	5.2
Delaware	216,513	82,174	36.5	5.6
Florida	4,695,524	69,303	34.3	11.2
Georgia	2,599,616	72,837	34.7	11.7
Hawaii	302,734	92,458	35.4	3.5
Idaho	436,965	72,785	32.3	8.2
Illinois	2,852,991	76,708	37.7	6.6
Indiana	1,530,011	66,785	29.6	7
Iowa	693,875	69,588	32.3	4.5
Kansas	649,589	68,925	35.6	8.6
Kentucky	988,972	59,341	27.9	5.6
Louisiana	1,043,544	55,416	27.1	6.9
Maine	282,427	69,543	36.1	6.6
Maryland	1,395,676	94,991	43.8	6.1
Massachusetts	1,610,759	94,488	46.6	2.4
Michigan	2,176,561	66,986	32.1	4.5
Minnesota	1,255,719	82,338	39.1	4.5
Mississippi	669,121	52,719	24.8	10.8
Missouri	1,368,368	64,811	32.2	8.6
Montana	239,382	67,631	34.6	8.3
Nebraska	435,512	69,597	34.7	6.7
Nevada	719,713	72,333	27	11.1
New Hampshire	290,658	89,992	41.3	4.9
New Jersey	2,039,450	96,346	43.5	6.8
New Mexico	464,654	59,726	30.5	8.2
New York	4,457,006	79,557	40	4.9
North Carolina	2,438,121	67,481	35.9	9.3
North Dakota	172,587	71,970	31.8	6.4
Ohio	2,567,828	65,720	32	5.9
Oklahoma	911,644	59,673	28.5	11.7
Oregon	960,141	75,657	36.3	6
Pennsylvania	2,804,185	71,798	35.1	5.3
Rhode Island	246,284	81,854	39.6	4.2
South Carolina	1,169,623	64,115	32.6	9.1
South Dakota	191,168	69,728	31.6	8.1
Tennessee	1,598,739	65,254	31.1	9.3
Texas	7,214,941	72,284	33.9	16.6
Utah	842,091	89,168	37.9	8.1
Vermont	137,539	73,991	44.2	3.9
Virginia	1,978,978	85,873	42.2	6.5
Washington	1,786,627	91,306	39.5	6.1
West Virginia	365,994	54,329	24.8	5.9
Wisconsin	1,269,086	70,996	33.2	5.2
Wyoming	124,653	70,042	29.6	11.5
United States	75,125,383	74,755	35.7	8

**Table 2.** Breastfeeding rates by state.

State	Initiation, %	3 months, %	6 months, %
Alabama	71.1	38	21
Alaska	92.9	57.6	30.9
Arizona	85.4	43.2	24
Arkansas	74.9	42	24.4
California	89.9	51.6	27.3
Colorado	94	62.8	32.1
Connecticut	84.2	44.7	26.3
Delaware	83.6	48.3	25
Florida	71	32.4	18.2
Georgia	82.6	39.9	18.7
Hawaii	90.1	50.6	27.7
Idaho	93.5	57.6	30.4
Illinois	84.9	47.8	28.3
Indiana	85.9	46.2	21.5
Iowa	82.4	52.8	27
Kansas	87.1	47	29.2
Kentucky	74.7	35.4	21.2
Louisiana	71.1	38	22.2
Maine	86.6	50.5	28.7
North Dakota	85.7	48.8	27.4
Massachusetts	80	52.8	29.2
Michigan	83.1	42.6	25.1
Minnesota	91.9	57.5	36.5
Mississippi	69.4	31.1	15.6
Missouri	78.3	42.5	24.6
Montana	83.5	50.4	34.3
Nebraska	86.1	49.3	26
Nevada	83.8	42.4	22.3
New Hampshire	82.2	55	31.8
New Jersey	82.5	41.2	23.5
New Mexico	83.4	52.3	29
New York	86.7	42.4	23.4
North Carolina	83.4	47.2	22.1
North Dakota	85.7	48.8	27.4
Ohio	79.5	42.7	23.7
Oklahoma	77.3	43.1	23.2
Oregon	87.2	59.2	34.2
Pennsylvania	74.8	42.4	24.6
Rhode Island	82.4	42.3	22.9
South Carolina	80.6	43.3	19.3
South Dakota	88.9	52.1	29.1
Tennessee	78.8	41.9	24.9
Texas	84.1	42.4	24
Utah	91.4	49.5	27.3
Vermont	91.8	61	36.2
Virginia	83.3	39.6	25.8
Washington	93.7	57	29.5
West Virginia	59.8	28	13.8
Wisconsin	87.5	59.3	31.3
Wyoming	92.4	55.3	27.2
United States	83.2	45.3	24.9

The initiation rates range from 59.8% in West Virginia to 94% in Colorado. Exclusivity rates at 3 months range from 28% in West Virginia to 62.8% in Colorado. Exclusivity rates at 6 months range from 13.8% in West Virginia to 36.5% in Minnesota. The number of IBCLCs and IBCLC density in each state are shown in Table 3.

**Table 3.** IBCLC^a^ density by state.

State	Total IBCLCs	Female childbearing population	IBCLCs per 100,000
Alabama	211	1,143,118	18.5
Alaska	99	163,121	60.7
Arizona	414	1,637,098	25.3
Arkansas	117	677,271	17.3
California	2640	9,124,204	28.9
Colorado	446	1,380,866	32.3
Connecticut	243	804,136	30.2
Delaware	59	216,513	27.3
Florida	788	4,695,524	16.8
Georgia	515	2,599,616	19.8
Hawaii	101	302,734	33.4
Idaho	135	436,965	30.9
Illinois	624	2,852,991	21.9
Indiana	470	1,530,011	30.7
Iowa	162	693,875	23.3
Kansas	210	649,589	32.3
Kentucky	167	988,972	16.9
Louisiana	195	1,043,544	18.7
Maine	85	282,427	30.1
Maryland	495	1,395,676	35.5
Massachusetts	458	1,610,759	28.4
Michigan	482	2,176,561	22.1
Minnesota	443	1,255,719	35.3
Mississippi	97	669,121	14.5
Missouri	360	1,368,368	26.3
Montana	65	239,382	27.2
Nebraska	144	435,512	33.1
Nevada	108	719,713	15.0
New Hampshire	95	290,658	32.7
New Jersey	514	2,039,450	25.2
New Mexico	135	464,654	29.1
New York	1057	4,457,006	23.7
North Carolina	778	2,438,121	31.9
North Dakota	33	172,587	19.1
Ohio	708	2,567,828	27.6
Oklahoma	210	911,644	23.0
Oregon	510	960,141	53.1
Pennsylvania	726	2,804,185	25.9
Rhode Island	60	246,284	24.4
South Carolina	263	1,169,623	22.5
South Dakota	42	191,168	22.0
Tennessee	312	1,598,739	19.5
Texas	1401	7,214,941	19.4
Utah	183	842,091	21.7
Vermont	82	137,539	59.6
Virginia	621	1,978,978	31.4
Washington	626	1,786,627	35.0
West Virginia	73	365,994	19.9
Wisconsin	368	1,269,086	29.0
Wyoming	18	124,653	14.4
United States	19,148	75,125,383	25.5

a IBCLC: International Board Certified Lactation Consultant.

The average IBCLC density for the entire United States female childbearing age population is 25.5, while the state with the highest density of IBCLCs was Alaska at 60.7 and the lowest density was in Wyoming at 14.4.

The simple linear regression for IBCLC density and breastfeeding initiation shown in Table 4 yielded a correlation coefficient of 0.38 with a *P* value of <.001 and an *R*^2^ of 0.26.

The simple linear regression for IBCLC density and exclusive breastfeeding at 3 months shown in Table 5 yielded a correlation coefficient of 0.52 with a *P* value of <.001 and an *R*^2^ of 0.41.

The simple linear regression for IBCLC density and exclusive breastfeeding at 6 months shown in Table 6 yielded a correlation coefficient of 0.32 with a *P* value of <.001 and an *R*^2^ of 0.41.

The multiple linear regression analysis for breastfeeding initiation shown in Table 7 has an adjusted *R*^2^ of 0.44, and it produced a coefficient of 0.26 for IBCLC density with a *P* value of .005. Income had an extremely low coefficient of 0.0003 with a *P* value of .005. Lack of health insurance coverage yielded a very high coefficient of 47.93, but a *P* value of .14. Having a bachelor’s degree or higher yielded a coefficient of −6.05 and *P* value of .82.

**Table 4.** Simple linear regression of IBCLC^a^ density and breastfeeding initiation.^b^

	Coefficient	SE	*t* test	*P*>|*t*|	95% CI
Constant	72.9925	2.5770	28.3250	<.001	67.8112-78.1739
IBCLCs per 100,000	0.3783	0.0891	4.2468	<.001	0.1992-0.5573

a IBCLC: International Board Certified Lactation Consultant.

b The parameters of the linear regression are described here. Model: ordinary least squares. Dependent variable: Initiation. Date: October 18, 2023. Number of observations: 50. Degrees of freedom of the model: 1. Residual degrees of freedom: 48. *R*2: 0.273. Adjusted *R*2: 0.258. Akaike information criterion: 324.9093. Bayesian information criterion: 328.7333. Log-likelihood: −160.45. *F* statistic: 18.04. Probability (*F* statistic): 9.89E-05. Scale: 37.38.

**Table 5.** Simple linear regression of IBCLC^a^ density and exclusive breastfeeding at 3 months.^b^

	Coefficient	SE	*t* test	*P*>|*t*|	95% CI
Constant	32.8530	2.5610	12.8283	<.001	27.7038-38.0022
IBCLCs per 100,000	0.5189	0.0885	5.8623	<.001	0.3409-0.6969

a IBCLC: International Board Certified Lactation Consultant.

b The parameters of the linear regression are described here. Model: ordinary least squares. Dependent variable: 3 months. Date: October 18, 2023. Number of observations: 50. Degrees of freedom of the model: 1. Residual degrees of freedom: 48. *R*2: 0.417. Adjusted *R*2: 0.405. Akaike information criterion: 324.2871. Bayesian information criterion: 328.1112. Log-likelihood: −160.14. *F* statistic: 34.37. Probability (*F* statistic): 4.07E-07. Scale: 36.918.

**Table 6.** Simple linear regression of IBCLC^a^ density and exclusive breastfeeding at 6 months.^b^

	Coefficient	SE	*t* test	*P*>|*t*|	95% CI
Constant	17.2583	1.5714	10.9830	<.001	14.0989-20.4178
IBCLCs per 100,000	0.3202	0.0543	5.8959	<.001	0.2110-0.4294

a IBCLC: International Board Certified Lactation Consultant.

b The parameters of the linear regression are described here. Model: ordinary least squares. Dependent variable: 6 months. Date: October 18, 2023. Number of observations: 50. Degrees of freedom of the model: 1. Residual degrees of freedom: 48. *R*2: 0.42. Adjusted *R*2: 0.408. Akaike information criterion: 275.4432. Bayesian information criterion: 279.2673. Log-likelihood: −135.72. *F* statistic: 34.76. Probability (*F* statistic): 3.61E-07. Scale: 13.899.

**Table 7.** Multiple regression for initiation.^a^

	Coefficient	SE	*t* test	*P*>|*t*|	95% CI
Constant	50.1350	6.9303	7.2342	<.001	36.1767 to 64.0934
IBCLCs^b^ per 100,000	0.2623	0.0882	2.9742	.005	0.0847 to 0.4399
Income	0.0003	0.0001	2.9213	.005	0.0001 to 0.0006
No health insurance	47.9309	31.9921	1.4982	.14	−16.5045 to 112.3662
Bachelor’s degree and higher	−6.0498	25.9169	−0.2334	.82	−58.2491 to 46.1496

a The parameters of the multiple regression are described here. Model: ordinary least squares. Dependent variable: initiation. Date: October 19, 2023. Number of observations: 50. Degrees of freedom of the model: 4. Residual degrees of freedom: 45. *R*2: 0.484. Adjusted *R*2: 0.438. Akaike information criterion: 313.7569. Bayesian information criterion: 323.317. Log-likelihood: −151.88. *F* statistic: 10.56. Probability (*F* statistic): 4.04E-06. Scale: 28.293.

b IBCLC: International Board Certified Lactation Consultant.

The results for exclusive breastfeeding at 3 months shown in Table 8 are as follows: adjusted *R*^2^=0.42, IBCLC density coefficient=0.43 with *P*<.001, income coefficient=0.0002 with *P*=.20, lack of health insurance coefficient=−8.17 with *P*=.82, and bachelor’s degree or higher coefficient=−0.62 with *P*=.98.

**Table 8.** Multiple regression for exclusive breastfeeding at 3 months.^a^

	Coefficient	SE	*t *test	*P*>|*t*|	95% CI
Constant	23.8639	7.7936	3.0620	.004	8.1667 to 39.5610
IBCLCs^b^ per 100,000	0.4282	0.0992	4.3175	<.001	0.2284 to 0.6279
Income	0.0002	0.0001	1.2987	.20	−0.0001 to 0.0004
No health insurance	−8.1700	35.9774	−0.2271	.82	−80.6322 to 64.2921
Bachelor’s degree or higher	−0.6175	29.1454	−0.0212	.98	−59.3194 to 58.0844

a The parameters of the multiple regression are described here. Model: ordinary least squares. Dependent variable: 3 months. Date: October 18, 2023. Number of observations: 50. Degrees of freedom of the model: 4. Residual degrees of freedom: 45. *R*2: 0.47. Adjusted *R*2: 0.423. Akaike information criterion: 325.4971. Bayesian information criterion: 335.0572. Log-likelihood: −157.75. *F* statistic: 9.996. Probability (*F* statistic): 7.10E-06. Scale: 35.781.

b IBCLC: International Board Certified Lactation Consultant.

Exclusive breastfeeding at 6 months results shown in Table 9 are as follows: adjusted *R*^2^=0.44, IBCLC density coefficient=0.25 with *P*<.001, income coefficient=0.0000 with *P*=.74, lack of health insurance coefficient=−15.34 with *P*=.49, and bachelor’s degree or higher coefficient=15.9 with *P*=.37.

**Table 9.** Multiple regression for exclusive breastfeeding at 6 months.^a^

	Coefficient	SE	*t* test	*P*>|*t*|	95% CI
Constant	12.7784	4.7405	2.6956	<.001	3.2306 to 22.3263
IBCLCs^b^ per 100,000	0.2537	0.0603	4.2054	.001	0.1322 to 0.3752
Income	0.0000	0.0001	0.3343	.74	−0.0001 to 0.0002
No health insurance	−15.3373	21.8834	−0.7009	.49	−59.4127 to 28.7381
Bachelor’s degree or higher	15.8958	17.7278	0.8967	.37	−19.8099 to 51.6015

a The parameters of the multiple regression are described here. Model: ordinary least squares. Dependent variable: 6 months. Date: October 18, 2023. Number of observations: 50. Degrees of freedom of the model: 4. Residual degrees of freedom: 45. *R*2: 0.482. Adjusted *R*2: 0.436. Akaike information criterion: 275.7809. Bayesian information criterion: 285.341. Log-likelihood: −132.89. *F* statistic: 9.996. Probability (*F* statistic): 4.40E-06. Scale: 35.7.

b IBCLC: International Board Certified Lactation Consultant.

## Discussion

### Principal Findings

This study found a positive association between the population density of IBCLCs and key breastfeeding outcomes in the United States, including initiation, exclusive breastfeeding at 3 months, and exclusive breastfeeding at 6 months (Multimedia Appendix 2). The strongest relationship was observed for exclusive breastfeeding at 3 months. These findings align with previous research showing that IBCLCs are associated with improved breastfeeding duration and exclusivity. For example, Patel and Patel [7] reported significant increases in breastfeeding rates when lactation support was provided in both hospital and community settings, and our findings reinforce the importance of IBCLC access beyond the point of care delivery. Similarly, Chrzan-Dętkoś et al [6] found that lactation consultations not only improved exclusivity but also maternal mental health, suggesting multifaceted benefits that may partly explain the state-level effects observed in our analysis.

Importantly, our study adds to this body of work by analyzing IBCLC density as a structural factor at the population level. Although Perrine et al [5] showed that hospital practices aligned with the Baby-Friendly Hospital Initiative improve exclusive breastfeeding intentions, our data demonstrate that widespread IBCLC availability across an entire state is similarly associated with improved breastfeeding metrics. This macro-level perspective complements prior individual-level studies and offers a broader lens on access equity.

These findings suggest that increased access to professional lactation support may play an important role in promoting breastfeeding continuation during the critical early months of infancy.

### Comparison to Prior Work

The association between IBCLC density and breastfeeding outcomes identified in this study is consistent with literature emphasizing the clinical effectiveness of professional lactation support. Previous studies have documented improvements in breastfeeding duration and maternal confidence following targeted lactation interventions [6,7]. However, these studies were conducted in localized or clinical settings. Our analysis broadens this understanding by showing that even at the population level, the presence of IBCLCs is significantly associated with improved breastfeeding outcomes, indicating that workforce distribution matters as much as clinical quality.

Additionally, Rollins et al [1] emphasized how gaps in health system structures and commercial pressures undermine breastfeeding support globally. Our findings offer a US-specific parallel, suggesting that one way to counteract these systemic barriers is to increase the geographic accessibility of IBCLCs. Unlike initiatives limited to single institutions, the presence of IBCLCs across regions may mitigate inequities in maternal health resources.

Furthermore, while Perrine et al [5] focused on hospital practices, we extend their insights by suggesting that system-level workforce planning (ie, ensuring every region has adequate access to IBCLCs) could serve as a complementary public health strategy. By embedding our results in this broader context, we highlight how both institutional practices and population-based interventions may be needed to achieve national breastfeeding goals.

### Strengths and Limitations

This study has several strengths. It draws from comprehensive, publicly available national datasets, applies both simple and multiple linear regression models, and includes key sociodemographic variables as potential confounders. This enhances the reliability of the observed associations and provides a foundation for future public health policy exploration.

However, several limitations must be acknowledged.

First, the study relies on secondary data sources, which limits the inclusion of other relevant variables such as cultural norms, maternity leave policies, or maternal health conditions. Attempts were made to address this through the inclusion of known confounders (income, education, and insurance status), but unmeasured variables likely remain.

Second, the focus on IBCLCs exclusively excludes other types of lactation support providers (eg, Certified Lactation Counselors; Women, Infants, and Children Peer Counselors), which may result in an underestimation of lactation support availability in some regions. This may particularly affect underserved areas where IBCLCs are sparse but alternative supports are present.

Third, the breastfeeding outcome data are based on parent-reported responses collected through telephone surveys, which may be susceptible to recall or social desirability bias. Although the CDC’s methods are well-established and designed to minimize such issues, this limitation is inherent in self-reported data.

Fourth, the analysis was conducted at the state level, which may obscure important regional disparities. Breastfeeding behaviors and IBCLC availability likely vary significantly within states, suggesting the need for future studies using data at the county or ZIP code level.

### Future Directions

To build on these findings, future research should focus on more granular geographic data, such as IBCLC access by zip code or county, to better identify disparities masked by state-level averages. Linking provider registries with localized breastfeeding surveillance systems may also enhance understanding of the real-world accessibility of lactation support. Additionally, expanding the analysis to include other types of lactation professionals and examining awareness of services among postpartum individuals could provide valuable insights. Longitudinal research tracking changes in IBCLC workforce density and breastfeeding rates over time would further clarify the nature of these relationships.

### Conclusions

Given that national breastfeeding rates remain subpar, widespread promotion of breastfeeding as the sociocultural norm is a paramount initiative. The statistical results of this research collectively indicate that IBCLC density plays a valuable role in promoting positive breastfeeding outcomes, even when considering potential confounding factors. The positive correlations between IBCLC density and breastfeeding rates at various time points are supported by the simple linear and multiple regression analyses, highlighting the significance of lactation consultants in achieving breastfeeding success. However, these findings should be interpreted in light of certain limitations, including the use of aggregated state-level data and exclusion of non-IBCLC lactation professionals. Future research would benefit from more granular geographic data, such as breastfeeding rates at the county or zip code level and IBCLC distribution. Such data would enable more precise correlation and may uncover regional disparities masked in state-level analysis. One approach to improve data granularity could involve integrating provider workforce registries with public health datasets at the substate level or collaborating with hospitals and health departments to collect real-time lactation support utilization data. Additionally, exploring longitudinal outcomes and incorporating combination feeding practices could enhance the ecological validity of future studies. Many mothers are not aware that they can receive comprehensive health care from a lactation consultant to prevent and solve breastfeeding complications. Even with a fair population density of IBCLCs in an area, not having an awareness of their services poses a barrier to that care. Improved public awareness and policy-level initiatives that fund and integrate IBCLCs into postnatal care structures may significantly boost breastfeeding continuation rates. Further research with more granular baseline data would be an excellent start for promoting the expansion of IBCLC care and improving breastfeeding rates.

## Supplementary material

10.2196/70098 Multimedia Appendix 1 Definition of terms.

10.2196/70098 Multimedia Appendix 2 Sequentially sorted data: International Board Certified Lactation Consultants density and breastfeeding rates.

## Abbreviations

CDC: Centers for Disease Control and Prevention
IBCLC: International Board Certified Lactation Consultants
IBLCE: International Board of Lactation Consultant Examiners
STROBE: Strengthening the Reporting of Observational Studies in Epidemiology
