# Supplementary material for: Lactation Consultant Access and Breastfeeding Outcomes in the United States: Cross-Sectional Analysis
Source: Interact J Med Res. 2025 Jul 17;14:e70098. doi: 10.2196/70098 (PMC12288860; doi:10.2196/70098)
Supplement: Multimedia Appendix 1 [file ijmr-v14-e70098-s001.docx]

**Multimedia Appendix 1. Definition of Terms**

The following list includes terms used in the research:

- Any breastfeeding: breastfeeding with no determined frequency, duration, or amount; can include the use of artificial baby milk
- Artificial baby milk: breastmilk substitute, typically called infant formula
- Breastfeeding initiation: first feeding after birth was at the breast; disregards any future feedings beyond the first and whether feeding was successful; can also be referred to as “ever breastfed”
- Exclusive breastfeeding: breastfeeding without the use of other foods or liquids, with the exception of medications, vitamins, and supplements
- Lactation consultant: healthcare professional trained and certified in lactation and breastfeeding dyads
- International Board Certified Lactation Consultant: the gold standard of lactation professional credentials with a rigorous certification process; also the only clinical lactation certification
