# Supplementary material for: Lactation Consultant Access and Breastfeeding Outcomes in the United States: Cross-Sectional Analysis
Source: Interact J Med Res. 2025 Jul 17;14:e70098. doi: 10.2196/70098 (PMC12288860; doi:10.2196/70098)
Supplement: Multimedia Appendix 2 [file ijmr-v14-e70098-s002.docx]

**Table 10.** *Sequentially sorted data: IBCLC density and breastfeeding rates.*

| **STATE** | **IBCLC DENSITY** | **STATE** | **INITIATION** | **STATE** | **3 MONTHS** | **STATE** | **6 MONTHS** |  |  |
| --- | --- | --- | --- | --- | --- | --- | --- | --- | --- |
| Alaska | 60.7 | South Dakota | 94.0% | Colorado | 62.8% | Minnesota | 36.5% |  |  |
| Vermont | 59.6 | Virginia | 93.7% | Vermont | 61.0% | Vermont | 36.2% |  |  |
| Oregon | 53.1 | Pennsylvania | 93.5% | Wisconsin | 59.3% | Montana | 34.3% |  |  |
| Maryland | 35.5 | UNITED STATES | 92.9% | Oregon | 59.2% | Oregon | 34.2% |  |  |
| Minnesota | 35.3 | Alabama | 92.4% | Alaska | 57.6% | Colorado | 32.1% |  |  |
| Washington | 35.0 | Washington | 91.9% | Idaho | 57.6% | New Hampshire | 31.8% |  |  |
| Hawaii | 33.4 | Wyoming | 91.8% | Minnesota | 57.5% | Wisconsin | 31.3% |  |  |
| Nebraska | 33.1 | Indiana | 91.4% | Washington | 57.0% | Alaska | 30.9% |  |  |
| New Hampshire | 32.7 | Vermont | 90.1% | Wyoming | 55.3% | Idaho | 30.4% |  |  |
| Kansas | 32.3 | New York | 89.9% | New Hampshire | 55.0% | Washington | 29.5% |  |  |
| Colorado | 32.3 | Kansas | 88.9% | Iowa | 52.8% | Kansas | 29.2% |  |  |
| North Carolina | 31.9 | North Carolina | 87.5% | Massachusetts | 52.8% | Massachusetts | 29.2% |  |  |
| Virginia | 31.4 | Wisconsin | 87.2% | New Mexico | 52.3% | South Dakota | 29.1% |  |  |
| Idaho | 30.9 | Tennessee | 87.1% | South Dakota | 52.1% | New Mexico | 29.0% |  |  |
| Indiana | 30.7 | Massachusetts | 86.7% | California | 51.6% | Maine | 28.7% |  |  |
| Connecticut | 30.2 | Ohio | 86.6% | Hawaii | 50.6% | Illinois | 28.3% |  |  |
| Maine | 30.1 | Utah | 86.1% | Maine | 50.5% | Hawaii | 27.7% |  |  |
| New Mexico | 29.1 | Oregon | 85.9% | Montana | 50.4% | Maryland | 27.4% |  |  |
| Wisconsin | 29.0 | Florida | 85.7% | Utah | 49.5% | North Dakota | 27.4% |  |  |
| California | 28.9 | West Virginia | 85.7% | Nebraska | 49.3% | California | 27.3% |  |  |
| Massachusetts | 28.4 | Mississippi | 85.4% | Maryland | 48.8% | Utah | 27.3% |  |  |
| Ohio | 27.6 | Iowa | 84.9% | North Dakota | 48.8% | Wyoming | 27.2% |  |  |
| Delaware | 27.3 | Oklahoma | 84.2% | Delaware | 48.3% | Iowa | 27.0% |  |  |
| Montana | 27.2 | Georgia | 84.1% | Illinois | 47.8% | Connecticut | 26.3% |  |  |
| Missouri | 26.3 | Arizona | 83.8% | North Carolina | 47.2% | Nebraska | 26.0% |  |  |
| Pennsylvania | 25.9 | New Hampshire | 83.6% | Kansas | 47.0% | Virginia | 25.8% |  |  |
| UNITED STATES | 25.5 | Nevada | 83.5% | Indiana | 46.2% | Michigan | 25.1% |  |  |
| Arizona | 25.3 | North Dakota | 83.4% | UNITED STATES | 45.3% | Delaware | 25.0% |  |  |
| New Jersey | 25.2 | South Carolina | 83.4% | Connecticut | 44.7% | Tennessee | 24.9% |  |  |
| Rhode Island | 24.4 | Rhode Island | 83.3% | South Carolina | 43.3% | UNITED STATES | 24.9% |  |  |
| New York | 23.7 | Missouri | 83.2% | Arizona | 43.2% | Missouri | 24.6% |  |  |
| Iowa | 23.3 | Kentucky | 83.1% | Oklahoma | 43.1% | Pennsylvania | 24.6% |  |  |
| Oklahoma | 23.0 | Idaho | 82.6% | Ohio | 42.7% | Arkansas | 24.4% |  |  |
| South Carolina | 22.5 | Minnesota | 82.5% | Michigan | 42.6% | Arizona | 24.0% |  |  |
| Michigan | 22.1 | Maryland | 82.4% | Missouri | 42.5% | Texas | 24.0% |  |  |
| South Dakota | 22.0 | Michigan | 82.4% | Nevada | 42.4% | Ohio | 23.7% |  |  |
| Illinois | 21.9 | Texas | 82.2% | New York | 42.4% | New Jersey | 23.5% |  |  |
| Utah | 21.7 | Louisiana | 80.6% | Pennsylvania | 42.4% | New York | 23.4% |  |  |
| West Virginia | 19.9 | New Mexico | 80.0% | Texas | 42.4% | Oklahoma | 23.2% |  |  |
| Georgia | 19.8 | New Jersey | 79.5% | Rhode Island | 42.3% | Rhode Island | 22.9% |  |  |
| Tennessee | 19.5 | Hawaii | 78.8% | Arkansas | 42.0% | Nevada | 22.3% |  |  |
| Texas | 19.4 | Nebraska | 78.3% | Tennessee | 41.9% | Louisiana | 22.2% |  |  |
| North Dakota | 19.1 | Maine | 77.3% | New Jersey | 41.2% | North Carolina | 22.1% |  |  |
| Louisiana | 18.7 | Colorado | 74.9% | Georgia | 39.9% | Indiana | 21.5% |  |  |
| Alabama | 18.5 | Montana | 74.8% | Virginia | 39.6% | Kentucky | 21.2% |  |  |
| Arkansas | 17.3 | California | 74.7% | Alabama | 38.0% | Alabama | 21.0% |  |  |
| Kentucky | 16.9 | Connecticut | 71.1% | Louisiana | 38.0% | South Carolina | 19.3% |  |  |
| Florida | 16.8 | Delaware | 71.1% | Kentucky | 35.4% | Georgia | 18.7% |  |  |
| Nevada | 15.0 | Arkansas | 71.0% | Florida | 32.4% | Florida | 18.2% |  |  |
| Mississippi | 14.5 | Alaska | 69.4% | Mississippi | 31.1% | Mississippi | 15.6% |  |  |
| Wyoming | 14.4 | Illinois | 59.8% | West Virginia | 28.0% | West Virginia | 13.8% |  |  |
